# Structural Requirements for the Procoagulant Activity of Nucleic Acids

**DOI:** 10.1371/journal.pone.0050399

**Published:** 2012-11-30

**Authors:** Julia Gansler, Miriam Jaax, Silke Leiting, Bettina Appel, Andreas Greinacher, Silvia Fischer, Klaus T. Preissner

**Affiliations:** 1 School of Medicine, Institute of Biochemistry, Justus-Liebig-University, Giessen, Germany; 2 Institute for Immunology und Transfusion Medicine, Ernst-Moritz-Arndt-University, Greifswald, Germany; 3 Institute of Biochemistry, Ernst-Moritz-Arndt-University, Greifswald, Germany; Carl-Gustav Carus Technical University-Dresden, Germany

## Abstract

Nucleic acids, especially extracellular RNA, are exposed following tissue- or vessel damage and have previously been shown to activate the intrinsic blood coagulation pathway *in vitro* and *in vivo*. Yet, no information on structural requirements for the procoagulant activity of nucleic acids is available. A comparison of linear and hairpin-forming RNA- and DNA-oligomers revealed that all tested oligomers forming a stable hairpin structure were protected from degradation in human plasma. In contrast to linear nucleic acids, hairpin forming compounds demonstrated highest procoagulant activities based on the analysis of clotting time in human plasma and in a prekallikrein activation assay. Moreover, the procoagulant activities of the DNA-oligomers correlated well with their binding affinity to high molecular weight kininogen, whereas the binding affinity of all tested oligomers to prekallikrein was low. Furthermore, four DNA-aptamers directed against thrombin, activated protein C, vascular endothelial growth factor and nucleolin as well as the naturally occurring small nucleolar RNA U6snRNA were identified as effective cofactors for prekallikrein auto-activation. Together, we conclude that hairpin-forming nucleic acids are most effective in promoting procoagulant activities, largely mediated by their specific binding to kininogen. Thus, *in vivo* application of therapeutic nucleic acids like aptamers might have undesired prothrombotic or proinflammatory side effects.

## Introduction

For a long time, the (patho-) physiological role of the intrinsic pathway of blood coagulation remained unclear, largely because the initiation of the “contact phase” activation was thought to occur mainly via artificial surfaces such as kaolin or glass [1], [2]. Although fibrillar collagens or heparin were proposed to serve as natural promoters of contact phase activation (via factor XII autoactivation) [3], [4], recent studies from our and other laboratories proposed that extracellular nucleic acids, or polyphosphates and denatured proteins may serve as efficient cofactors in the onset of intrinsic pathway activation under patho-physiological conditions [5], [6], [7], [8]. In particular, being an “alarm signal” under conditions of tissue damage or vascular injury, extracellular nucleic acids were proposed as “natural foreign surface” to initiate factor XII autoactivation, resulting in enhanced thrombus formation [5]. Several *in vivo* models confirmed the involvement of extracellular RNA in arterial and venous thrombus formation, edema formation and vascular hyperpermeability. Interestingly enough, administration of ribonuclease 1 (RNase 1) significantly reduced or prevented thrombus formation, stroke and development of edema in respective animal models [5], [9]. Under conditions of activation of innate immunity with the formation of neutrophil extracellular traps (NET), such DNA-histone networks were shown to provide a procoagulant surface as well [8].

Mechanistically, structure-function relationships that were considered as determining factors for the procoagulant activity of extracellular nucleic acids include: (a) the high negative charge density of nucleic acids and (b) their polyanionic nature, required for high affinity binding to the proteins of the contact phase (including factors XII and XI, high molecular weight kininogen and prekallikrein) [5]. In addition, (c) sufficiently long extracellular RNA was found to promote protease activation in this system via a “template mechanism”, by which both, the respective protease and its protein substrate need to bind to the same RNA molecule, which then may catalyse protease conversion [5], [10]. Although additional factors and conditions may contribute to the functional activity of procoagulant nucleic acid molecules, a structure-based analysis of their respective functions has not been undertaken so far.

In the present study we provide first evidence that the secondary structure of DNA and RNA oligonucleotides appears to be important for their function as cofactors of the intrinsic coagulation pathway *in vitro*. To this end, the influence of length, secondary structure and sequence of such DNA- and RNA-oligomers was assessed by functional coagulation assays. Furthermore, we addressed the question, whether artificial nucleic acids in form of DNA-aptamers, which recently provided promising results as therapeutics for several diseases, were able to promote contact phase activation. Additionally, we analyzed the procoagulant activity of small nucleolar RNAs (snRNAs), which are released in protein complexes during systemic lupus erythematosus (SLE), an auto-immune disease with prevalent thrombotic side-effects [11], [12].

Our results indicate that small, double-stranded nucleic acid compounds, including four DNA-aptamers and a snRNA, were most effective to serve as promoters of intrinsic blood coagulation, very likely through specific binding of the cofactor protein kininogen.

## Materials and Methods

### Material

**Figure 1:**
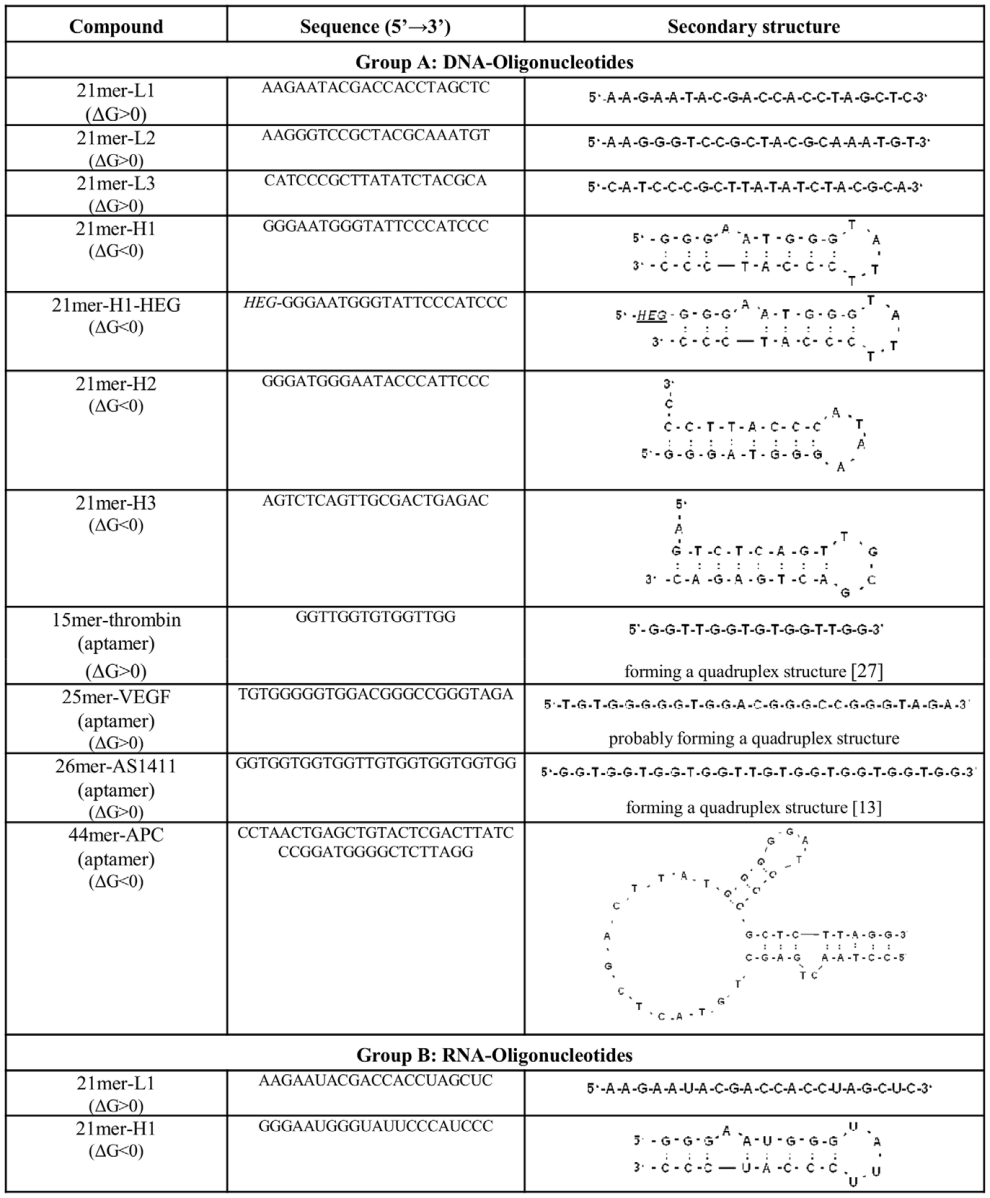
Sequences and secondary structures of DNA- and RNA-oligonucleotides. Described are the secondary structures of (A) DNA- and (B) RNA-oligonucleotides as predicted by the mFold DNA or RNA database. Delta G (ΔG) values represent changes in free enthalpy representative for the stability of the compounds with negative (exergonic) or positive values (endergonic).

RNA-oligonucleotides were purchased from Purimex (Grebenstein, Germany); biotinylated, unlabeled and hexaethylenglycol-modified DNA-oligonucleotides were from Biomers (Ulm, Germany). Sequences of the tested RNA and DNA constructs used in this study are given in figure 1. Secondary structures of RNA- and DNA-oligonucleotides were calculated by the mFold RNA and mFold DNA database of the University of Albany (USA). Poly (I:C) was purchased from InvivoGen (San Diego, USA). Chromogenic substrate S2366, single-chain high molecular weight kininogen and tissue plasminogen activator were from Haemochrom Diagnostica (Essen, Germany); prekallikrein, factor XI and factor XII were from American Diagnostica (Pfungstadt, Germany). RNase, DNase, 2× RNA loading dye, 6× DNA loading dye and 100 bp DNA ladder and the restriction enzyme BamH1 with according reaction buffer were from Fermentas (St. Leon-Rot, Germany). PureLink HiPure Plasma Midiprep Kit, Quant-iT™ RNA assay kit and Qubit fluorometer were from Invitrogen (Carlsbad, USA). DNA-plasmid and reagents for *in vitro* transcription of U6snRNA were provided by Dr. Albrecht Bindereif (Institute for Biochemistry, Justus-Liebig-University, Giessen, Germany).

### *In vitro* Transcription of U6snRNA

U6snRNA-containig DNA-plasmid was amplified and purified by using the PureLink HiPure Plasma Midiprep Kit according to the manufacturer’s instructions, followed by digestion with the restriction enzyme BamH1 and purification via phenol/chloroform extraction. RNA-transcription of 1 µg BamH1-digested DNA-plasmid was performed at 37°C for one hour by using SP6 polymerase buffer, 10 mM dithiothreitol, 0.5 mM of the ribonucleotides adenosine-, cytosine- and uracile-triphosphate as well as 0.1 mM guanosine-triphospate, 20 units RNase Inhibitor and 30 units of SP6-polymerase. Remaining DNA was digested by incubation with 2 units of DNase at 37°C for 30 min. RNA was isolated by phenol/chloroform extraction. The quality of the RNA-transcript was confirmed by agarose gel electorphoresis followed by staining in a 20% (v/v) ethidium bromide solution. Concentration of RNA was measured with the Quant-iT™ RNA assay kit and the Qubit fluorometer.

### Plasma Preparation

Blood samples were obtained from the blood bank, University Hospital, Giessen. All patients had to give a written consent, approved by the Ethics Committee of the Medical Faculty, Justus-Liebig-University, Giessen, file number 05/00. Data were analyzed anonymously. Blood samples were centrifuged at 2,800×g at 4°C for 10 min to obtain platelet-poor plasma, pooled and aliquots were frozen at −80°C until further use.

### Polyacrylamide Gel Electrophoresis of RNA- and DNA-oligonucleotides

Oligonucleotides were subjected to electrophoresis (1.25 µg/lane) onto 20% (v/v) polyacrylamide gels and seperated using tris-borate-EDTA buffer, followed by ethidium bromide staining. For digestion experiments, 1.25 µg of the respective oligonucleotides was incubated for 1 or 5 min at 37°C with 10 µL of pooled human EDTA- plasma, followed by addition of 10 µL RNA loading dye or 1.5 µL DNA loading dye and subsequent polyacrylamide gel electrophoresis. Gels were stained in a 20% (v/v) ethidium bromide solution.

### Clot/lysis Assay in Human Plasma

A 96-well microtiter plate was blocked with 3% (w/v) bovine serum albumin (BSA) in Tris-buffered saline (TBS) at 37°C for 1 h. All reagents to be added were dissolved in 0.1 M imidazol to a final volume of 100 µL. Increasing concentrations of RNA- or DNA-oligonucleotides were incubated with 100 ng/mL tissue plasminogen activator (tPA). After addition of pooled EDTA-plasma the plate was incubated for 5 min at 37°C. Following addition of CaCl_2_ (200 nM) to start the reaction, clotting times, defined as time points with maximal absorbance, were recorded up to 30 min at 405 nm by using KC4 software (BIO-TEK, Bad Friedrichshall, Germany).

### Prekallikrein Acitivity Assay

A 96-well microtiter plate was blocked with 3% (w/v) BSA in HEPES buffered saline (HBS) at 37°C for 1 h. All reagents were dissolved in HBS to a final volume of 100 µL. Twenty nM prekallikrein was incubated with 55 nM high molecular weight kininogen, 0.3 mM chromogenic substrate S2366, 50 µM ZnCl_2_ and increasing concentrations of U6snRNA, poly (I:C) or artificial RNA- and DNA-oligonucleotides, respectively. Prekallikrein (auto-) activation was registered up to 60 min by cleavage of chromogenic substrate S2366 and recorded at 405 nm by using KC4 software (BIO-TEKs).

### Binding of Nucleic Acids to Proteins

Biotinylated DNA-oligonucleotides were purchased from Biomers (Ulm, Germany). Microtiter plate wells were coated with 50 µL solutions of prekallikrein, high molecular weight kininogen, factor XI or factor XII (10 µg/mL each) in 100 mM sodium carbonate (pH 9.5) at 4°C for 20 h. Wells coated with 100 mM sodium carbonate (pH 9.5) only were used as blank for detection of unspecific binding of biotinylated DNA-oligonucleotides. Wells were washed and blocked with TBS containing 3% (w/v) BSA for 2 h. Different concentrations of biotinylated DNA-oligonucleotides (0.31–50 µg/mL) were allowed to bind to adsorbed proteins at 22°C for 2 h followed by three times washing with TBS. Bound biotinylated nucleic acids were detected using peroxidase-conjugated streptavidin (Dako, Glostrup, Denmark) and the immunopure TMB (3,3′,5,5′-tetramethylbenzidine) substrate kit (Pierce, Rockford, USA) by quantitating the reaction products at 450 nm.

### Statistics

Results were expressed as the mean ± standard error of the mean (SEM). Two-paired analysis of variance (ANOVA) and subsequent multiple comparison using Bonferroni-post test were used for statistical analysis. Results were considered as significant at p<0.05.

## Results

### Stability of RNA- and DNA-oligonucleotides in Plasma

**Figure 2:**
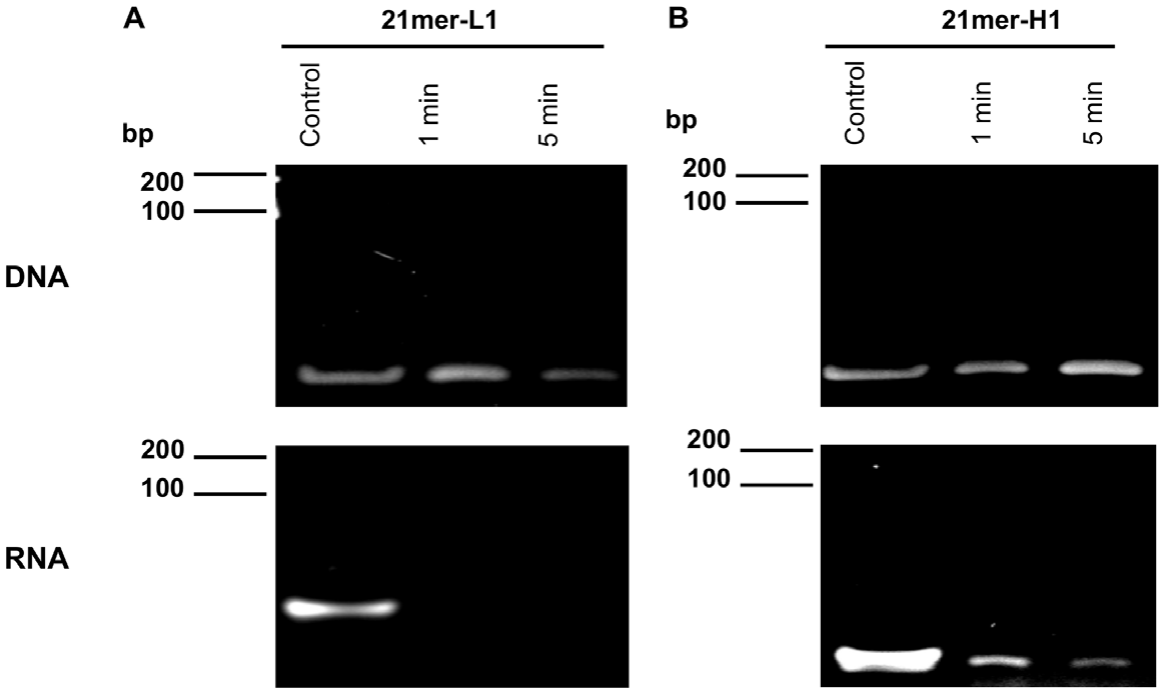
Stability of DNA- and RNA-oligonucleotides in human plasma. (A) Integrity of 21mer-L1 and (B) 21mer-H1 DNA- and RNA-oligonucleotides was confirmed by polyacrylamide gel electrophoresis without or after preincubation in pooled human plasma for 1 or 5 min, respectively. Each panel represents one representative experiment out of three independent ones.

Cellular RNA and DNA have been demonstrated to serve as cofactors for the initiation of the intrinsic pathway of coagulation by promoting the auto-activation of the contact phase proenzymes [5]. In order to gain insight into the structural features for their mode of action, two oligonucleotides forming different secondary structures (single-stranded, hairpin) were tested for their pro-coagulant cofactor function in plasma and in purified systems (Fig. 1). Firstly, the stability of these DNA- and RNA-oligonucleotides in human plasma was analyzed by polyacrylamide gel electrophoresis. Pretreatment with human plasma for 1–5 min revealed that all DNA-oligonucleotides remained largely stable, while the 21mer-L1 RNA-oligonucleotide immediately disappeared, likely due to hydrolysis. The 21mer-H1 RNA-oligonucleotide was only partly degraded during the time of incubation, and remained detectable even after 20 min (data not shown) due to higher stability in human plasma compared to the linear RNA-oligomer (Fig. 2A, 2B).

### Procoagulant Activity of RNA- and DNA-oligonucleotides

**Figure 3:**
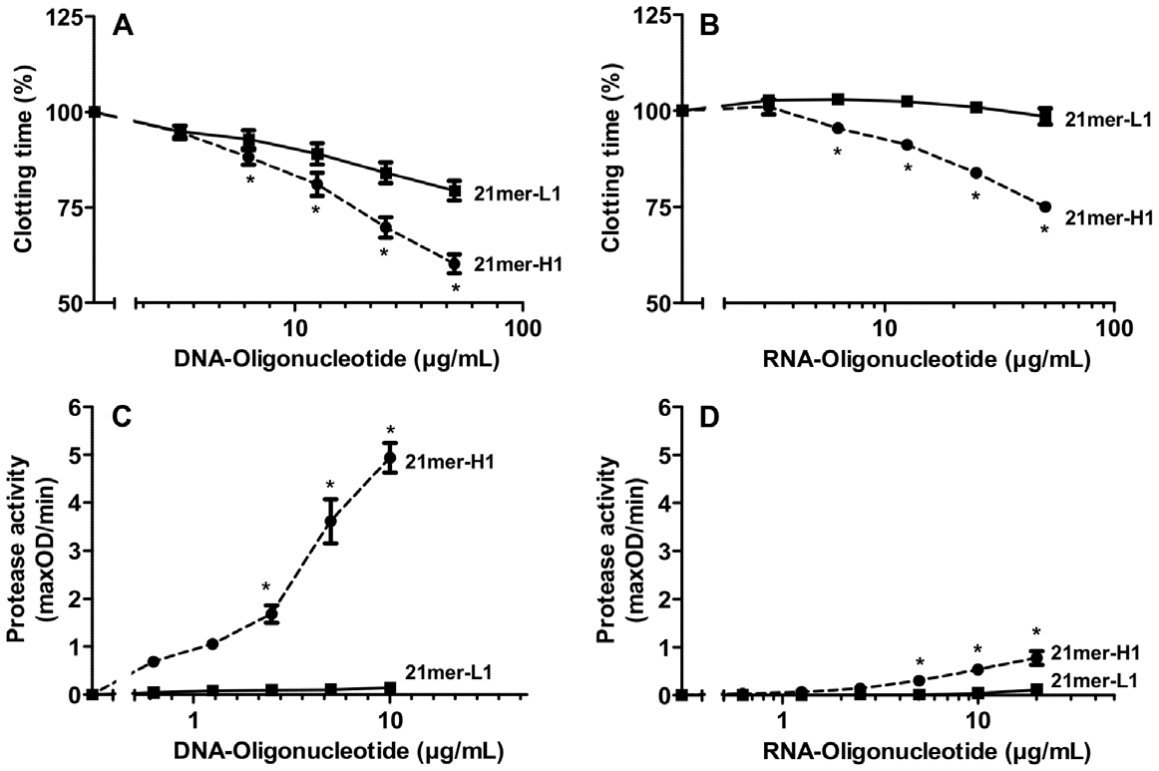
Procoagulant activity of DNA- and RNA-oligonucleotides with different length and secondary structures. Increasing concentrations of (A) DNA-oligonucleotides 21mer-H1 (closed circles) or 21mer-L1 (closed squares) as well as (B) the respective RNA-oligonucleotides, 21mer-H1 (closed circles) or 21mer-L1 (closed squares) were tested for procoagulant activity in a turbidity clot-lysis assay using pooled human plasma. Coagulation was initiated by recalcification; clotting times were defined as respective time points of maximal absorbance. The clotting time of untreated plasma was defined as 100%. The activation of prekallikrein was followed in the presence of increasing doses of (C) DNA-oligonucleotides 21mer-H1 (closed circles) or 21mer-L1 (closed squares) as well as (D) the respective RNA-oligonucleotides 21mer-H1 (closed circles) or 21mer-L1 (closed squares). Enzyme activity was registered by chromogenic substrate assay as described in “Material and Methods”. All data represent mean ± SEM (n≥3; *p<0.05; 21mer-H1 vs. 21mer-L1).

In a plasma recalcification clotting assay different concentrations of RNA- and DNA-oligonucleotides were tested for procoagulant activity. Among the DNA- and RNA-oligonucleotides, the 21mer-H1 was most effective to shorten the clotting time in a dose-dependent manner to 60% or 75% of the control value, respectively. The 21mer-L1-oligonucleotide promoted only a weak procoagulant effect (Fig. 3A, 3B).

To investigate the influence of different oligonucleotides on a typical protease activation step of the intrinsic coagulation pathway, prekallikrein auto-activation (in the presence of kininogen) was analysed. Among all RNA- and DNA-oligonucleotides, the 21mer-H1 DNA compound exhibited the most impressive cofactor activity in promoting prekallikrein auto-activation. No cofactor activity was observed for both 21mer-L1-oligonucleotides (Fig. 3C, 3D).

### Functional Activities of Different DNA-hairpin Structures

As the 21mer-H1 DNA oligonucleotide revealed the highest cofactor activity to promote coagulation, the procoagulant functions of different single-stranded as well as hairpin-forming DNA-oligonucleotides were compared (Fig. 1).

**Figure 4:**
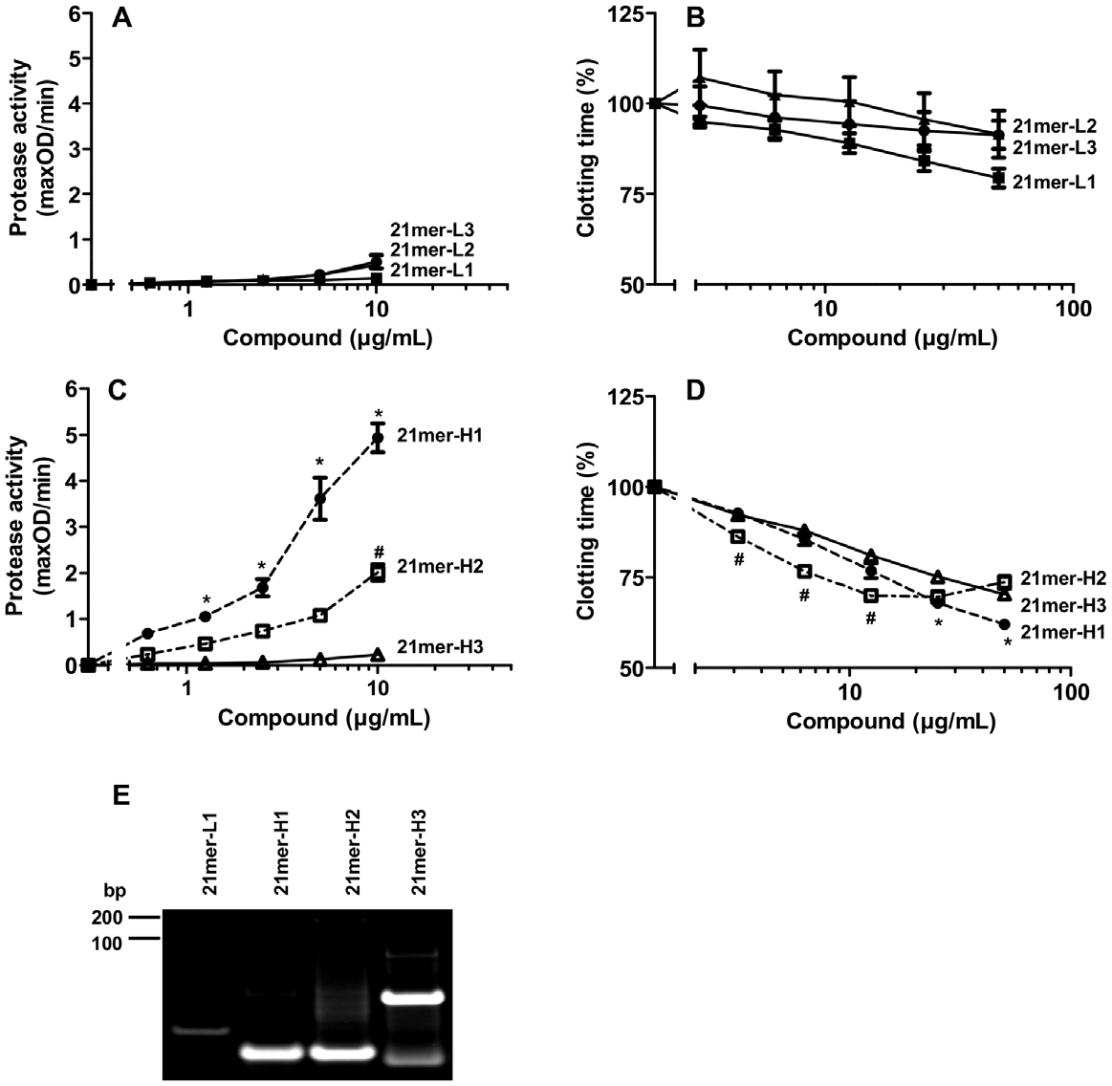
Procoagulant activity of different linear and hairpin-forming DNA-oligonucleotides. Increasing concentrations of the linear DNA-oligonucleotides 21mer-L1 (closed squares), 21mer-L2 (closed triangels) or 21mer-L3 (closed circles) were analyzed for (A) prekallikrein auto-activation or (B) procoagulant activity in a turbidity clot-lysis assay. The clotting time of untreated plasma was defined as 100%. All data represent mean ± SEM (n = 3). Increasing concentrations of the hairpin-forming DNA-oligonucleotides 21mer-H1 (closed circles), 21mer-H2 (open squares) or 21mer-H3 (open triangles) were analyzed for (C) prekallikrein auto-activation or (D) procoagulant activity in a turbidity clot-lysis assay. The clotting time of untreated plasma was defined as 100%. All data represent mean ± SEM (n≥3; *p<0.05; 21mer-H1 vs. 21mer-H3; #p<0.05; 21mer-H2 vs. 21mer-H3). (E) The sizes of DNA-oligonucleotides were analyzed by polyacrylamide gel electrophoresis. Shown is one representative experiment out of three independent ones.

All tested single-stranded DNA-oligonucleotides revealed no or only low effects in turbidity clot/lyis assays (Fig. 4B) or prekallikrein activity tests (Fig. 4A). In contrast, the blunt-ended 21mer-H1 exhibited prominent cofactor activity in promoting the auto-activation of prekallikrein, while 21mer-H2, which reveals a 3′-overhang, was considerably less active. Moreover, 21mer-H3, demonstrating a 5′-overhang, had no cofactor activity (Fig. 4C). All three compounds shortened the clotting time of human plasma in a dose-dependent manner, whereby 21mer-H1 and 21mer-H2 showed most prominent effects (Fig. 4D). As demonstrated by polyacrylamide gel electrophoresis, the DNA-oligonucleotides 21mer-H1 and 21mer-H2 represent single hairpin secondary structures, while the majority of 21mer-H3 may form dimers (Fig. 4E).

**Figure 5:**
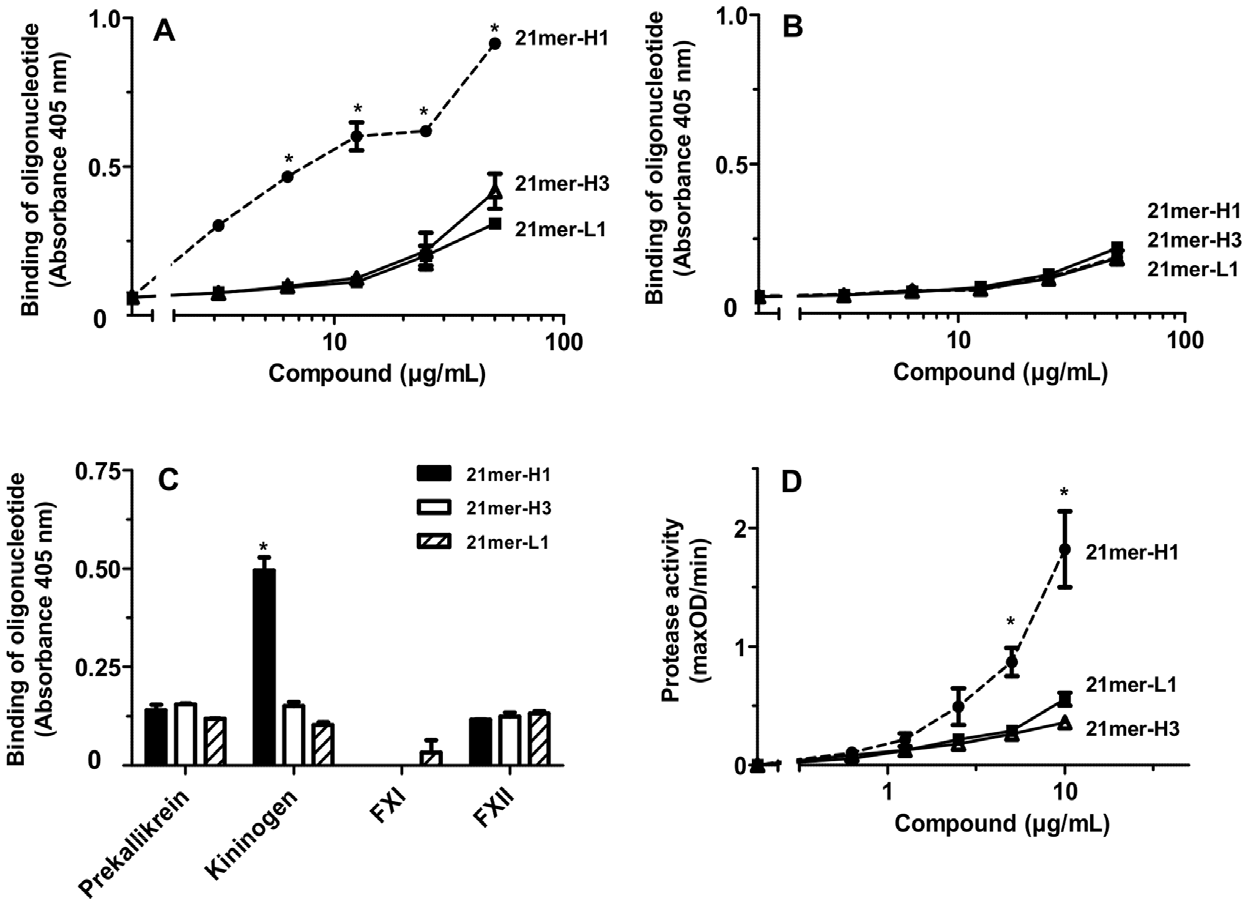
Binding of biotinylated DNA-oligonucleotides to different coagulation factors of the intrinsic coagulation pathway. Microtiter plate wells were coated with 10 µg/mL each of (A) kininogen or (B) prekallikrein and binding of increasing concentrations of the biotinylated DNA-oligonucleotides 21mer-H1 (closed circles), 21mer-H3 (open triangles), 21mer-L1 (closed squares) was assessed. All data represent mean ± SD (n = 3; *p<0.05; 21mer-L1 and 21mer-H3 vs. 21mer-H1) of one representative experiment out of three independent ones. (C) Microtiter plate wells were coated with 10 µg/mL kininogen, factor XI (FXI) or factor XII (FXII) each and incubated with 25 µg/mL each of different biotinylated DNA-oligonucleotides: 21mer-H1 (black bars), 21mer-H3 (white bars) or 21mer-L (hatched bars). All data represent mean ± SD (n = 3) of one representative experiment out of three independent ones. (D) Increasing concentrations of the biotinylated DNA-oligonucleotides 21mer-H1 (closed circles), 21mer-H3 (open triangles) or 21mer-L1 (closed squares) were analyzed for prekallikrein auto-activation. All data represent mean ± SEM (n≥3; *p<0.05; 21mer-H1 vs. 21mer-H3).

In order to corroborate these findings, binding assays using biotinylated forms of two 21mer-DNA-hairpins as well as the 21mer-L1-molecule were performed. The oligonucleotide 21mer-H1 revealed most efficient binding to kininogen with a dissociation constant of K_D_ = 1.82 µM (±0.23) (Fig. 5A). All tested DNA-oligomers only showed weak binding interactions to prekallikrein (Fig. 5B). Furthermore, all tested oligomers exhibited low binding to factor XII, while binding towards factor XI remained undetectable (Fig. 5C). Functionality of the biotinylated oligonucleotides was confirmed by prekallikrein activity assays (Fig. 5D).

### Contact Phase Activation by DNA-aptamers

**Figure 6:**
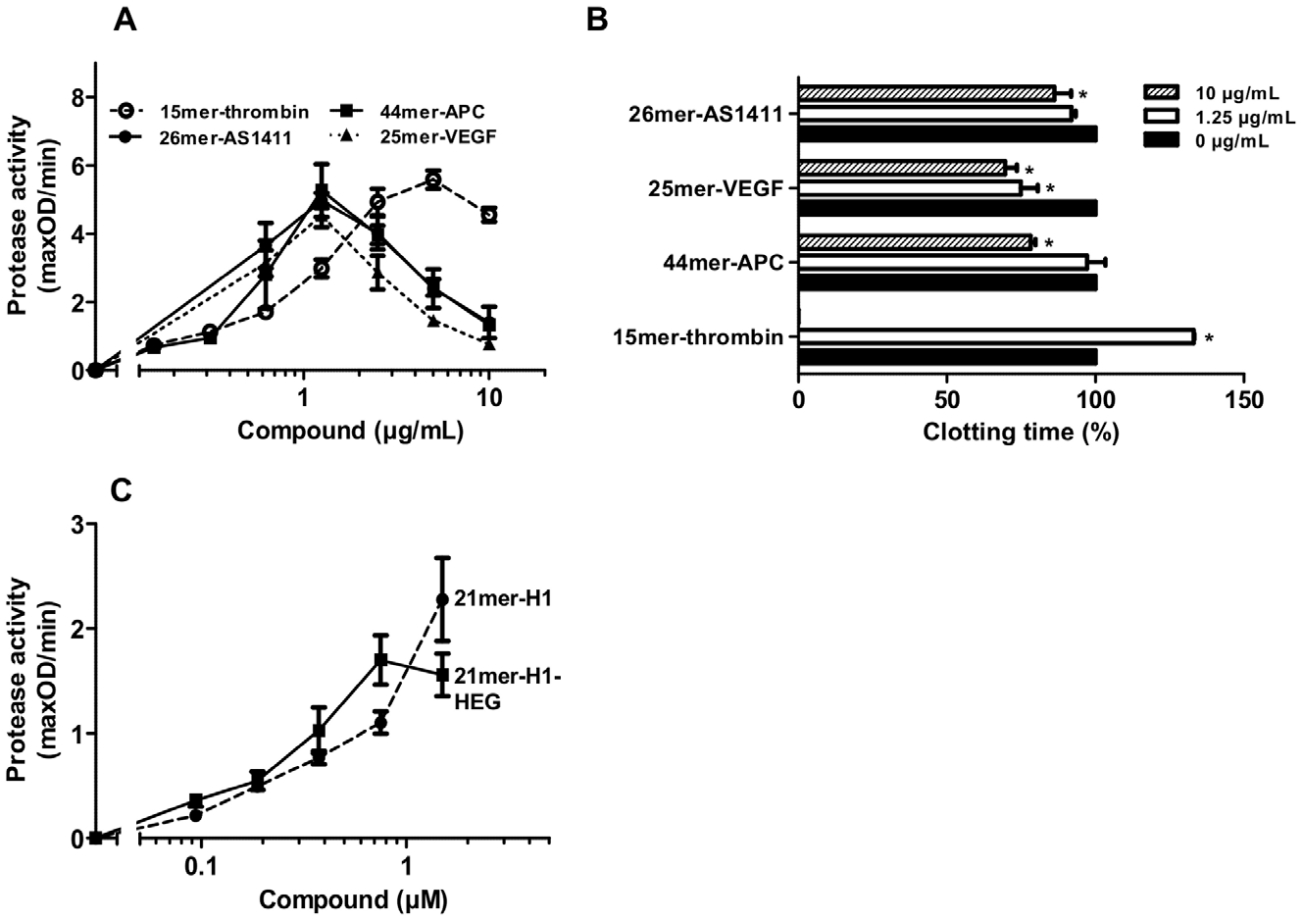
Influences of DNA-aptamers on the intrinsic coagulation pathway. (A) The activation of prekallikrein was followed in the presence of increasing doses of the DNA-aptamers 15mer-thrombin (open circles, interrupted line), 44mer-APC (closed squares), 26mer-AS1411 (closed circles) or 25mer-VEGF (closed triangles, dotted line). (B) Turbidity clot-lysis assays were performed in the absence (black bars) or presence of 1.25 µg/mL (white bars) and 10 µg/mL (striped bars) of the DNA-aptamers 15mer-thrombin, 44mer-APC, 26mer-AS1411 or 25mer-VEGF, respectively. Coagulation was initiated by recalcification, clotting times were defined as respective time points of maximal absorbance. The clotting time of untreated plasma was defined as 100%. All data represent mean ± SEM (n = 3; *p<0.05; 1.25 µg/mL or 10 µg/mL vs. control). (C) The activation of prekallikrein was followed in the presence of increasing doses of the oligonucleotide 21mer-H1 (closed circles) and 21mer-H1-HEG (closed squares). All data represent mean ± SEM (n = 6).

Aptamers, exhibiting complex secondary structures, which promote specific binding to target proteins, were analyzed for their procoagulant functions. Therefore, four different apatmers that are known as potent inhibitors of thrombin, activated protein C (APC), vascular endothelial growth factor (VEGF) or nucleolin (AS1411) were chosen (Fig. 1) [13], [14], [15], [16]. All aptamers were identified as efficient procoagulant cofactors by triggering the auto-activation of prekallikrein, whereby the 15mer-thrombin aptamer revealed the lowest effects (Fig. 6A). As the cofactor activity of all tested aptamers decreased at higher concentrations, a template mechanism is most likely. The functionality of the 15mer-thrombin aptamer to inhibit thrombin formation in human plasma was confirmed, as the clotting time was significantly prolonged or completely impaired at high concentrations of the aptamer. In contrast, all other tested aptamers promoted a significant reduction of the clotting time (Fig. 6B).

As most aptamers with potential clinical relevance are chemically modified to improve their stability in plasma, the influence of one possible modification on the procoagulant function of DNA-oligonucleotides was analyzed. Therefore a 5′-hexaethylenglycol was introduced to the 21mer-H1 DNA-oligonucleotide and prekallikrein auto-activation was measured. No significant difference was observed between the modified and the unmodified compound. (Fig. 6C).

### Procoagulant Activity of Small Nucleolar RNA (snRNA)

**Figure 7:**
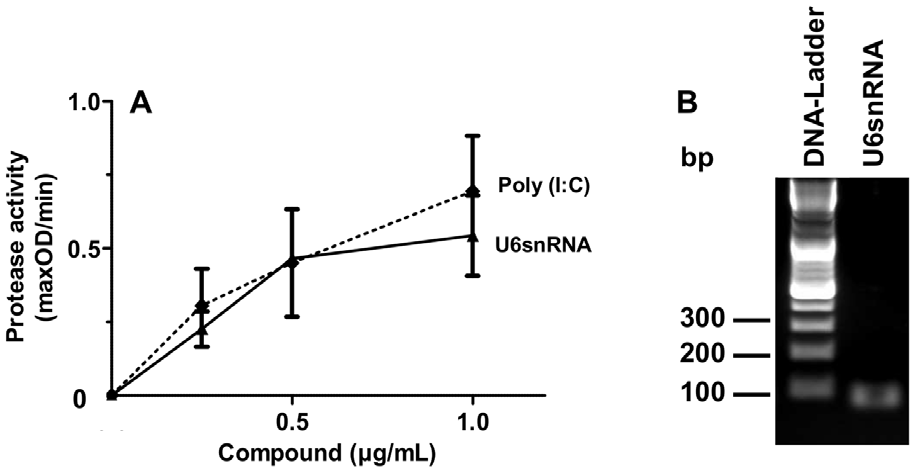
Procoagulant activity of snRNAs. (A) Increasing concentrations of U6snRNA (closed triangles, black line) or poly (I:C) (closed diamonds, dotted line) were analyzed for prekallikrein auto-activation. All data represent mean ± SEM (n = 3). (B) Integrity of U6snRNA was confirmed by agarose gelelectrophoresis.

Spliceosomal protein-RNA complexes (small nuclear ribonucleoprotein particles, snRNPs) serve as auto-antigens in several auto-immune diseases like systemic lupus erythematosus (SLE), which is often accompanied by thrombotic side-effects [11]. To address the question, whether the protein-associated snRNA can contribute to a procoagulant state, the influence of purified U6snRNA (Fig. 7B) on prekallikrein auto-activation was determined. U6snRNA promoted the auto-activation of prekallikrein in a concentration-dependent manner, which was comparable in its degree to the artificial RNA-polymer poly (I:C) (Fig. 7A).

## Discussion

Our study describes structural features of nucleic acids that are required for promoting procoagulant cofactor function. Three different aspects were identified to influence procoagulant activities of small nucleotides *in vitro*: stability, secondary structure and multimerisation. The analysis of stability of DNA- and RNA-oligomers in human plasma demonstrated that all DNA-oligomers remained largely intact confirming former studies which revealed that DNA-oligomers are stable in plasma for up to two hours independently of their structure [17]. In contrast, the decay rate of RNA-oligomers, largely due to hydrolysis by RNases which are present in human plasma [18], [19], appeared to be dependent on the secondary structure of the compounds. Accordingly, our results demonstrate that linear RNA-structures are hydrolyzed immediately, while hairpin-forming compounds are more stable and were detectable for up to 20 min in human plasma. Moreover, a clear correlation was seen between secondary structure-dependent stability of nucleic acid oligomers and functional activity as procoagulant cofactors.

To further analyze the interactions between nucleic acid oligomers and coagulation processes, the binding abilities of different DNA-oligonucleotides to coagulation proteins were compared. The data revealed that only the 21mer-H1 DNA oligomer specifically interacted with high molecular weight kininogen, whereas the other tested compounds presented low or no binding to kininogen, prekallikrein, FXI or FXII. High molecular weight kininogen is a multifunctional protein that serves to accelerate the activation of FXII by kallikrein as well as the activation of FXI [20], [21], [22] by forming non-covalent equimolar complexes either with FXI [23], [24] or with prekallikrein in the presence of zinc ions [20], [24], [25], [26]. Eighty percent of prekallikrein is bound to kininogen in plasma, and this complex may become associated with negatively charged surfaces (like RNA or DNA) during contact phase activation [1], [2], [3], [4]. Our previous and present data indicate that contact phase activation is mediated via a specific binding of hairpin-structured nucleic acid oligomers to kininogen, mediating activation of prekallikrein and FXI [5].

The present results extend the knowledge on differential interactions of nucleic acids with the blood clotting cascade. Until now, the roles of natural extracellular RNA and DNA as cofactors for the (auto-) activation of serine proteases in the initiation of the intrinsic coagulation pathway were mainly ascribed to charge-dependent interactions. Based on the present results, nucleic acids containing hairpin-forming structures provide potent procoagulant functions. These structures are present in naturally occurring RNA-macromolecules like ribosomal RNA, transfer-RNA and messenger RNA, which are exposed/released from the cytoplasm of damaged cells. Due to the presence of RNases in the circulation, the half-life of such RNA-species may be limited and therefore a systemic effect on activation of the clotting cascade would be moderate. Nevertheless, local RNA concentrations at the site of injury may be sufficient to trigger procoagulant effects.

Our study firstly identified a hairpin-containing, small nucleolar RNA (U6snRNA; 107 nucleotides), which is part of the spliceosome, as an efficient cofactor for the auto-activation of prekallikrein. Several studies already described elevated plasma levels of antibodies directed against snRNA-protein complexes (snRNPs) [12], [27] in patients suffering from SLE, an auto-immune disease that is accompanied by thrombotic side-effects with a prevalence of more than 10% (for review see [11]). Our results indicate that snRNAs, likely to be released due to abnormal apoptotic processes during SLE (for review see [28]), could contribute to this prothrombotic state already at very low concentrations. This effect was comparable to the activity of the artificial RNA-polymer poly (I:C), which has no defined secondary structure and promotes coagulation most likely via charge-dependent interactions due to its polyanionic character.

*In vivo* models of thrombosis and stroke revealed that pretreatment with RNase but not with DNase completely abolished procoagulant activities of extracellular RNA [5], [9]. This indicates that the physiological role of cellular DNA for activation of the clotting cascade under conditions of tissue damage is low, possibly due to its buried position within the nucleus and its association with histones [5], [9]. Additional studies of Oehmcke et al. identified extracellular DNA released by neutrophils under inflammatory conditions in form of neutrophil extracellular traps (NET) as physiologically relevant inducer of the intrinsic coagulation pathway [8]. This NET-forming DNA reveals stable secondary structures [29], which could, beside charge-dependent interactions, represent possible binding sites for serine proteases.

Our observations that hairpin-forming DNA-oligomers were most potent activators of the intrinsic coagulation pathway may also have clinical relevance, since activation of blood coagulation might be a potential side effect during the application of various DNA- or RNA-aptamers. First indications that DNA-aptamers have a procoagulant potential were presented in a study from Paul et al. in 2010, whereby DNA-aptamer libraries, which represent a mixture of DNA molecules with random sequences and secondary structures, were shown to activate the contact phase protein prekallikrein in an *in vitro* Chandler-Loop-model [30]. Their study, however, did not approach the question, whether all molecules of the library were able to provide cofactor functions or if only aptamers with certain characteristics were responsible for the described effects.

To address this question, the procoagulant functions of four DNA-aptamers against thrombin (determined to be anti-coagulant) [13], APC (determined to inhibit the anti-coagulant function of APC) [14], VEGF (binding to VEGF) [16] and AS1411 (inhibiting nucleolin function for cancer therapy) [15] were analyzed at concentrations comparable to plasma levels achieved during different clinical studies [31], [32], [33]. All aptamers revealed appreciable procoagulant cofactor function concerning the auto-activation of prekallikrein. Referring to the results discussed above, especially the already described quadruplex-structure of the thombin-aptamer, the AS1411-aptamer [15], [34], the probable quadruplex structure (due to high GC-content) of the VEGF-aptamer as well as the hairpin-containing sequences of the APC-aptamer should be considered as procoagulant domains.

Additionally, all aptamers except the thombin-aptamer reduced the clotting time of human plasma significantly, underlining their direct procoagulant function. As the significant increase in clotting time in human plasma confirmed the functionality of the thrombin-aptamer due to efficient inhibition of clot formation, no thrombotic side effects are expected by a systemic application of this compound. However, due to the activation of prekallikrein and the related release of bradykinin from kininogen, all tested aptamers could induce vasodilation or proinflammatory effects, which should be investigated by further studies. Preliminary experiments indicated a VEGF-mediated induction of endothelial hyperpermeability by different DNA-oligomers, which was independent of their secondary structure (data not shown). This effect was comparable to the already described RNA-induced hyperpermeability [9].

Furthermore, it has to be mentioned that the DNA-aptamers used for this study were not chemically modified. In contrast, most of the aptamers developed for therapeutical issues may carry several modifications including 5′-polyethylenglycol, 2′-fluoro-, 2′-aminomethyl- or 2′-O-methyl groups [35], [36], [37] to increase their stability in plasma. These modifications could modulate the here identified procoagulant activities of aptamers. To address this question, the procoagulant activity of the unmodified 21mer-H1 oligonucleotide was compared with a 21mer-H1 construct carrying a 5′-hexaethylenglycol modification. Both compounds revealed comparable promotion of auto-activation of prekallikrein, indicating that a 5′-hexaethylenglycol modification is not influencing the procoagulant activity of this DNA-olionucleotide.
